# K-Space Data Reconstruction Algorithm-Based MRI Diagnosis and Influencing Factors of Knee Anterior Cruciate Ligament Injury

**DOI:** 10.1155/2022/1711456

**Published:** 2022-06-01

**Authors:** Rui Chang, Angang Chen, Xiang Li, Xiaoqiang Song, Benqiang Zeng, Liping Zhang, Wanying Deng

**Affiliations:** ^1^Department of Orthopaedics, The People's Hospital of Wenjiang, Chengdu 611130, Sichuan, China; ^2^Department of Neurorehabilitation, Affiliated Sichuan Provincial Rehabilitation Hospital of Chengdu University of TCM, Chengdu 611130, Sichuan, China

## Abstract

This study was aimed at investigating the diagnostic value of MRI based on K-space data reconstruction algorithm for anterior cruciate ligament (ACL) injury of knee joint and the influencing factors of ligament injury. 96 patients with ACL injury of knee joint were selected, and they were randomly divided into two groups: group A (arthroscopy) and group B (MRI examination), and another 96 healthy volunteers in the same period were selected as the control group. The test results of each indicator were compared. The results showed that the signal-to-noise ratio (SNR) of SMASH algorithm was higher than that of sum of squares (SOS) algorithm. In group A, there were 66 positive and 30 negative tests, and in group B, there were 56 positive and 40 negative tests (*P* < 0.05). The intercondylar fossa width, the intercondylar fossa width index, and the ratio of tibial intercondylar eminence width to intercondylar fossa width in group B were lower than those in the control group (*P* < 0.05). Compared with the traditional SOS algorithm, SMASH algorithm can improve the image quality, reduce the impact of damage data on the final synthesis image, and improve the image SNR. In clinical work, the ratio of the width of tibial intercondylar eminence to the width of femoral intercondylar fossa can be measured by imaging data to evaluate the matching between tibial intercondylar eminence and femoral intercondylar fossa, so as to evaluate the risk of ACL rupture.

## 1. Introduction

Anterior cruciate ligament (ACL) is the main component of the knee joint, one of the largest and most complex joints in the human body. It can effectively prevent excessive tibial forward movement and maintain the normal function of the knee joint under the combined action of other tissues of the knee joint [1]. Therefore, ACL injury of knee joint and meniscus injury of knee joint are the most common [2]. If the function and characteristics of ACL are examined from the perspectives of biomechanics, human anatomy, and human kinematics, it will be found that ACL injury presents considerable complexity and variability from the aspects of combined injury, injury mechanism, and sick population [3]. Without timely diagnosis and treatment after ACL injury, complications such as knee joint rotation instability, knee meniscus injury, and traumatic arthritis may occur, which may affect the normal function of the knee joint of patients and cause serious problems in daily life. In addition, the swelling and pain of the knee joint at the early stage of injury may interfere with the clinical examination and diagnosis [4]. It is difficult to accurately determine the severity or type of ACL injury, especially on the premise of rapid diagnosis and timely treatment.

The clinical diagnosis of ACL injury is mostly based on three progressive levels [5]. First, the clinical attending physician relies on face-to-face understanding of the patient's injury history and on-site physical examination to make a preliminary diagnosis. There are many inspection methods in physical examination. Generally, the physical examination experiments include the anterior drawer test, the Lachman test, and the pivot-shift test [6]. The second is the implementation of auxiliary examination methods. Arthrography, ultrasonography, and other examinations have been used to diagnose ACL injury in different historical periods, but they have not become the mainstream means due to their invasiveness, sensitivity, specificity, and accuracy [7]. Since the 1980s, MRI technology, which has been applied to the imaging diagnosis of knee soft tissue injury, has become one of the preferred imaging methods for initial diagnosis with its noninvasive, fast, powerful multiplanar tomography and high differentiation of soft tissue [8]. The recent development of related technologies has also significantly promoted the diagnostic application of MRI; in particular, some recent studies have shown that the progress of technology has made MRI more refined and accurate in the degree of fault segmentation [9]. Arthroscopy, as the “gold standard” of soft tissue injury such as ACL of knee joint, has great diagnostic advantages of intuitive and accurate diagnosis and clear qualification and positioning, but after all, it cannot become the preferred method as an invasive operation [10].

MRI technology uses the characteristics of nuclear spin motion to obtain the morphological images of human tissues, being widely used in clinical diagnosis. However, magnetic resonance imaging (MRI) needs long time, so it cannot meet the requirements of fast imaging such as brain function imaging and cardiac dynamic imaging [11]. However, parallel MRI uses the spatial position information of the phased array coil instead of the number of phase encoding steps, which can greatly improve the imaging speed and ensure the image quality and high spatial resolution [12]. The sum of squares (SOS) algorithm is considered to be the optimal image synthesis method without knowing the exact sensitivity of each phased array coil. However, the SOS algorithm uses the same weight to synthesize the images of each coil and cannot suppress the external noise well, resulting in the problems of signal deviation and low signal-to-noise ratio (SNR) of the final image [13]. Therefore, simultaneous acquisition of spatial harmonics (SMASH) reconstruction algorithm was introduced in detail as a representative of parallel imaging algorithm based on K-space domain. The smoothing filter was used to denoise the reconstructed image of each phased array coil, and then the sensitivity of each coil was used as the weight of image synthesis [14].

Therefore, by arthroscopic diagnosis, MRI based on K-space SMASH reconstruction algorithm was used for imaging diagnosis of patients. The application effect was explored by comparing the detection results. In addition, compared with the healthy population, the possible influencing factors in knee ligament injury were explored to provide data support for the prevention and diagnosis of knee cruciate ligament injury in clinical practice.

## 2. Research Methods

### 2.1. Research Objects

In this study, 96 patients with ACL injury of knee joint in hospital from September 2018 to September 2020 were randomly selected, with unlimited age and gender. A total of 96 patients, 44 males and 52 females, were finally included. They were randomly divided into two groups: group A (arthroscopy) and group B (MRI examination), and another 96 healthy volunteers in the same period were selected as the control group. All the objects signed informed consent, and this study had been approved by the ethics committee of hospital.

Inclusion criteria are as follows: clinical tests have at least one of the positive signs of the three basic tests (anterior drawer test, Lachman test, and pivot-shift test) and have a history of trauma and clinical symptoms such as joint swelling and pain. Exclusion criteria are as follows: patients with clear history of knee joint, such as tuberculous arthritis, rheumatoid arthritis, intra-knee tumor, or knee surgery.

### 2.2. Examination Methods

#### 2.2.1. Clinical Examination

In the anterior drawer test, the patient was in a supine position. The knee flexion was 90°, and the hip flexion was 45°. The examiner fixed the patient's feet and pulled the patient's lower legs from posterior to anterior by holding the upper end of the lower legs with both hands.

In the Lachman test, knee flexion was 15°; the examiner seized the lower end of the patient's femur and pulled the upper end of the tibia from posterior to anterior with the other hand. The anterior drawer test and Lachman test were graded according to the following criteria: degree I: the tibial anteversion of the injured side increased by 1–5 mm and had a good termination point compared with the healthy side; degree II: the gap between the two sides was 6–10 mm, with flexible termination point; degree III: the gap was greater than 10 mm, without termination point.

In the Pivot-shift test (MacIntosh method), the patient was in supine position; the examiner placed one hand on the lateral side of the patient's knee, grasped the heel with the other hand to internally rotate the lower leg, everted the knee, and gradually flexed the knee joint from 0° position. When the affected knee was out of the buttoning-locking position, the lateral condyle of the tibia began to be gradually subluxated anteriorly, and when the knee was slowly flexed up to about 30°, the tibia suddenly reduced posteriorly and there was a sense of dislocation. The positive graduation of the test is as follows: degree I was slippage, degree II was dislocation, degree III was temporary interlocking. The clinical examination part was only used as preliminary screening, so it was only judged to be positive and negative.

There was at least one positive sign; then, it could be classified as a suspected case, and the second step of the examination was performed, that is, MRI examination.

#### 2.2.2. MRI Examination

An MRI machine and an SE sequence were used for relevant conventional scans.

The MRI findings of complete ACL injury include direct and indirect signs. The direct signs were interrupted ACL continuity, irregular and wavy ACL shape, abnormally increased signal in and around the ligament, no normal hypointense ligament fibers seen, reduced ACL inclination, and no parallel walking to the Blumensaat line. Indirect signs included posterior cruciate ligament curvature greater than 0.39, bone contusion on the posterolateral surface of the tibial plateau, depression of the lateral femoral notch greater than 1.5 mm, anterior displacement of tibial greater than 5 mm, and posterior displacement of the posterior horn of the lateral meniscus. According to the MRI findings of partial ACL injury, the ligament morphology was normal, while localized abnormal signals appeared in the ligament; some ligament fibers were curved or wavy.

### 2.3. Measurement of Relevant Anatomical Parameters

Measurement of relevant anatomical parameters of femoral intercondylar notch was performed using 3.0T MRI scanner, with slice thickness of 2 mm. The patient was placed in supine position. The knee joint was naturally extended in non-weight-bearing state. The sagittal, coronal, and axial scans of the knee joint were performed. On coronal images, the level at which the medial and lateral femoral condyles maintained continuity and the popliteal groove could be observed was selected as the level at which the intercondylar notch width index was measured. The intercondylar notch width index = the width of the intercondylar notch at the level of the popliteal groove/the width of both condyles at the same level.

Measurement of anatomical parameters related to tibial intercondylar augmentation was carried out as follows: the patient was placed in the supine position with the knee joint naturally extended in a non-weight-bearing state, and X-ray examination was performed on the knee joint. The height of the medial and lateral apices of the tibial intercondylar eminence and the width of the tibial intercondylar eminence were measured on intact anteroposterior radiographs of the knee. First, the connecting line between the most concave point of the medial tibial plateau and the most convex point of the lateral tibial plateau was used as the joint line, and vertical lines were made from the apices of the medial and lateral tibial intercondylar eminence to the joint line. The length of these two vertical lines was the medial height of the tibial intercondylar eminence and the lateral height of the tibial intercondylar eminence, and the distance between the two vertical lines and the intersection point of the joint line was the width of the tibial intercondylar eminence.

### 2.4. Parallel Imaging Reconstruction Algorithm Based on K-Space Domain

K-space is Fourier space, also known as spatial frequency space or raw data space, which is the filling space of the original data of magnetic resonance signal with spatial positioning coding information [15].

For two-dimensional K-space, the unit is spatial frequency, which is expressed by the number of cycles cm^−1^ or *H*_*z*_·cm^−1^, and is described by two mutually perpendicular vectors, *K*_*x*_, *K*_*y*_; that is, K-space is the space defined by the two coordinate components *K*_*x*_, *K*_*y*_. The line with *K*_*y*_=0 is called zero Fourier line, the Fourier line close to *K*_*y*_=0 is called low spatial frequency Fourier line, and that far away from *K*_*y*_=0 is called high spatial frequency Fourier line. Thus, the K-plane region can be divided into low-frequency Fourier space and high-frequency Fourier space.

The conversion relationship between K-space and data space is in a uniform applied magnetic field; the magnetic resonance signal is directly proportional to the transverse magnetization vector, but in a nonuniform applied magnetic field space or in the presence of gradient magnetic field, the magnetic resonance signal is related not only to the proton spin density, but also to its spatial position. It is supposed that the vector form of the two-dimensional space coordinate where the sample is located, is $\underset{r}{⟶}=\left(x,y\right)$; the proton spin density in unit space is $\rho \left(\underset{r}{⟶}\right)=\rho \left(x,y\right)$; the time of repetition (TR) is long enough; and the time of echo (TE) is short enough. The expression of magnetic resonance signal *S*(*t*) is as follows:

(1) $$\begin{array}{c} S\left(t\right)=\int \rho \left(\underset{r}{⟶}\right)e^{-i2\pi \gamma \int_{0}^{t}G\left(t^{\prime }\right)\text{d}t^{\prime }}d^{2}\underset{r}{⟶}. \end{array}$$

In (1), *γ* is the magnetogyric ratio, and *G*(*t'*) is the gradient field. K-space is $\underset{K}{⟶}=\underset{K}{⟶}\left(t\right)=\gamma \int_{0}^{t}G\left(t^{\prime }\right)\text{d}t^{\prime }$; then, the following equation can be obtained:

(2) $$\begin{array}{c} S\left(\underset{K}{⟶}\right)=\int \rho \left(\underset{r}{⟶}\right)e^{-i2\pi K\left(t\right)d^{2}\underset{r}{⟶}}. \end{array}$$

**Figure 1:**
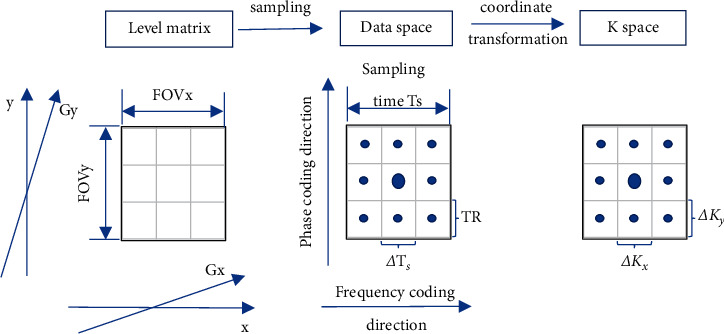
Process from level matrix to K-space.

Under the condition of $\underset{K}{⟶}=\underset{K}{⟶}\left(t\right)=\gamma \int_{0}^{t}G\left(t^{\prime }\right)\text{d}t^{\prime }$, the proton spin density represented as $\rho \left(\underset{r}{⟶}\right)$ of the object in the original coordinate $\underset{r}{⟶}$ and the acquired signal represented as $S\underset{K}{⟶}$ of the object in K-space are Fourier transform pair: $S\underset{K}{⟶}$ is the Fourier transform of $\rho \left(\underset{r}{⟶}\right)$; $\rho \left(\underset{r}{⟶}\right)$ is the inverse Fourier transform of $S\underset{K}{⟶}$. Therefore, the original coordinate $\underset{r}{⟶}$ with unit as cm will correspond to the spatial frequency coordinate $\underset{K}{⟶}$ with unit as *H*_*z*_·cm^−1^.  Figure 1 shows the process from level matrix to K-space. In Figure 1, *x* and *y* represent frequency coding and phase coding, FOV_*x*_ and FOV_*y*_ are the field of vision in *x* and *y* directions, and Δ*T*_*s*_ is the sampling interval. Δ*K*_*x*_ and Δ*K*_*y*_ are the intervals of K-space along the *x* and *y* directions.

In (2), $\underset{K}{⟶}$ and $\underset{r}{⟶}$ are two-dimensional vectors, so the gradient field corresponding to the two coordinate components in K-space in MRI technology can be expressed as follows:

(3) $$\begin{array}{c} \underset{K}{⟶}=\underset{K}{⟶}\left(t\right)=\left[K_{x}\left(t^{\prime }\right),K_{y}\left(t^{\prime }\right)\right]. \end{array}$$

The component form is as follows:

(4) $$\begin{array}{c} K_{x}=K_{x}\left(t^{\prime }\right)=\gamma \int_{0}^{t}G_{x}\left(t^{\prime }\right)dt\;\prime ,\\ K_{y}=K_{y}\left(t^{\prime }\right)=\gamma \int_{0}^{t}G_{y}\left(t^{\prime }\right)dt\;\prime . \end{array}$$

For the case where the gradient field change rate is constant, (4) can be expressed as follows:

(5) $$\begin{array}{c} K_{x}=\gamma G_{x}t,\\ K_{y}=\gamma G_{y}t. \end{array}$$

The conversion equation of converting the coordinates of data space into spatial frequency domain can be deduced as follows:

(6) $$\begin{array}{c} \Delta K_{x}=\frac{\gamma G_{x}\Delta T_{s}}{2\pi }=\frac{1}{{\text{FOV}}_{x}},\\ \Delta K_{y}=\frac{\gamma G_{y}\Delta T_{s}}{2\pi }=\frac{1}{{\text{FOV}}_{y}}. \end{array}$$

Parallel imaging based on K-space domain uses the K-space data collected by each phased array coil and the fitted weighting coefficient to recover the unsampled data in K-space, obtain the full K-space data of each phased array coil, and then carry out inverse Fourier transform to reconstruct the final image of each phased array coil [16]. SMASH reconstruction algorithm was introduced as a representative of parallel imaging algorithm based on K-space domain.

**Figure 2:**
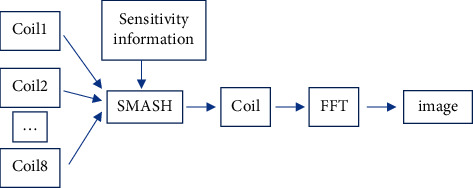
Schematic diagram of SMASH.

The SMASH reconstruction algorithm mainly uses the linear combination of phased array coil sensitivities to recover the K-space phase coded line data lost due to undersampling [17], and its brief schematic diagram is shown in Figure 2.

It is supposed that there is a group of phased array coils, each phased array coil has its unique sensitivity *S*_*i*_(*x*, *y*), and a mixed sensitivity *S*_*m*_^comp^(*x*, *y*) can be obtained through the linear combination of coils. The linear combination equation is as follows:

(7) $$\begin{array}{c} S_{m}^{\text{comp}}\left(x,y\right)=\sum_{i=1}^{N_{c}}w_{i}^{\left(m\right)}S_{i}\left(x,y\right). \end{array}$$

In (7), *m* is the ordinal number of spatial harmonics. *m*=−*R*/2, −*R*/2+1,…0,1,…*R*/2, *w*_*i*_^(*m*)^ are the weight coefficients of the *i* coil to the *m* harmonic. *N*_*c*_ is the number of coils forming the array. It is supposed that the weight coefficient *w*_*i*_^(*m*)^ and coil sensitivity *S*_*i*_(*x*, *y*) are as follows:

(8) $$\begin{array}{c} \sum_{i=1}^{N_{c}}w_{i}^{\left(m\right)}S_{i}\left(x,y\right)\approx e^{-2\pi jm\Delta k_{y}y}. \end{array}$$

The only unknown *w*_*i*_^(*m*)^ in (8) is calculated by *S*_*i*_(*x*, *y*) merging with *e*^*jm*Δ*k*_*y*_*y*^.

It is supposed that *I*_*i*_(*k*_*x*_, *k*_*y*_) is the K-space data collected by the *i* coil; then, the result of Fourier transform after sensitivity coefficient *S*_*i*_(*x*, *y*) weighting by the proton spin density *ρ*(*x*, *y*)  is as follows:

(9) $$\begin{array}{c} I_{i}\left(k_{x},k_{y}\right)=\int \int S_{i}\left(x,y\right)\rho \left(x,y\right)e^{-2\pi j\left(k_{x}y+k_{y}y\right)}\text{d}x\text{d}y. \end{array}$$

In two-dimensional MRI, the magnetic resonance signal detected by the receiving coil can be described as the following equation in Fourier space.

(10) $$\begin{array}{c} I\left(k_{x},k_{y}\right)=\int \int \rho \left(x,y\right)e^{-2\pi j\left(k_{x}y+k_{y}y\right)}\text{d}x\text{d}y. \end{array}$$

*I*_*i*_(*k*_*x*_, *k*_*y*_) is weighted by *w*_*i*_^(*m*)^; the phase coded line data lost in K-space can be obtained.

(11) $$\begin{array}{c} I_{m}^{\text{comp}}\left(k_{x},k_{y}\right)=\sum_{i=1}^{N_{c}}w_{i}^{\left(m\right)}I_{i}\left(k_{x},k_{y}\right), \end{array}$$

(12) $$\begin{array}{c} I_{m}^{\text{comp}}\left(k_{x},k_{y}\right)=\sum_{i=1}^{N_{c}}\int \int S_{i}\left(x,y\right)\rho \left(x,y\right)e^{-2\pi j\left(k_{x}y+k_{y}y\right)}\text{d}x\text{d}y \end{array}$$

Finally, the following equation is obtained:

(13) $$\begin{array}{c} I_{m}^{\text{comp}}\left(k_{x},k_{y}\right)=I\left(k_{x},k_{y}+m\Delta k_{y}\right). \end{array}$$

Equations (11)–(13) show that the linear combination of data collected by each coil can be used to generate K-space displacement, which is similar to the traditional phase coding method using gradient magnetic field. The advantage of this is that the increase of data used for fitting improves the accuracy and robustness of linear weight coefficient estimation, and several additional rows of autocalibration signal (ACS) lines located in the center of K-space can be used for image reconstruction, which will improve the quality of reconstructed image.

### 2.5. Evaluation Method of Reconstructed Image

The goal of MRI is to obtain medical images with minimum error. According to the methods used to evaluate the quality of image synthesis methods from different principles and entry points, it can be divided into two classical categories, namely, qualitative analysis criteria and quantitative analysis criteria. The performance of the algorithm was measured through the qualitative analysis of reconstructed image comparison and the two quantitative evaluation algorithms of SNR and error image (ERR).

SNR mainly reflects the image quality through the ratio of signal intensity to noise intensity. It is an important index to measure the image quality. Its calculation equation is as follows:

(14) $$\begin{array}{c} \text{SNR}=20\times \mathrm{lg}\frac{\sum_{x,y}\left|I_{\text{rec}}\left(x,y\right)\right|}{\sum_{x,y}{\left|I_{\text{rec}}\left(x,y\right)-I_{\text{ref}}\left(x,y\right)\right|}^{2}}. \end{array}$$

*I*_rec_ is the reconstructed image, and *I*_ref_ is the reference image.

ERR mainly reflects the proximity between the reconstructed image and the reference image in the form of the absolute value of the difference. It intuitively reflects the reconstruction accuracy of the algorithm.

(15) $$\begin{array}{c} \text{ERR}=\sum_{j=1}^{N_{x}}\sum_{i=1}^{N_{y}}\left|I_{i,j}^{\text{rec}}\left(x,y\right)-I_{i,j}^{\text{ref}}\left(x,y\right)\right|. \end{array}$$

*I*_rec_(*x*, *y*) represents the reconstructed image, *I*_ref_(*x*, *y*) represents the reference image, and *N*_*x*_ and *N*_*y*_ are the number of pixels.

### 2.6. Statistical Methods

SPSS 22.0 software was used. The measurement data were expressed by independent sample *t*-test, and the counting data were expressed by *χ*^2^ test. The difference was statistically meaningful with *P* < 0.05. The number of true positive cases refers to the number of ACL injuries diagnosed by arthroscopy or MRI. The number of false positive cases refers to the number of ACL injuries diagnosed by arthroscopy or MRI. The number of true negative cases refers to the number of ACL injuries diagnosed by arthroscopy or MRI. The number of false negative cases refers to the number of ACL injuries diagnosed by arthroscopy or MRI.

## 3. Results

### 3.1. Image Reconstruction Result

**Figure 3:**
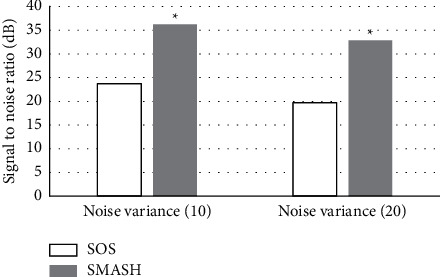
SNR of images synthesized by different algorithms. ^*∗*^Compared with SOS algorithm, *P* < 0.05.

The SNR of SMASH was higher than that of SOS. According to the definition of SNR, the larger the SNR, the higher the tissue signal strength and image clarity. This suggested that SMASH algorithm could improve image quality and had superiority (Figure 3).

**Figure 4:**
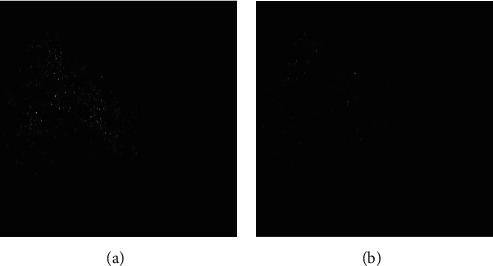
Difference image of algorithms. (a) SOS algorithm. (b) SMASH algorithm.

**Figure 5:**
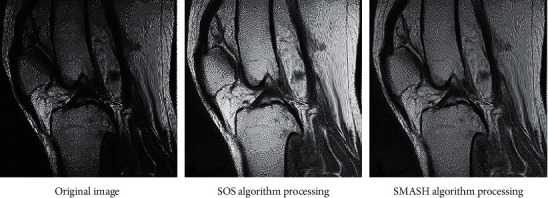
Image comparison of knee cruciate ligament injury.

Similarly, the difference image between the synthesized image and the reference image of the two algorithms was simulated (Figure 4). By comparison, there was almost no background noise in the difference image between the synthesized image and the reference image of the SMASH algorithm, and the ERR was almost invisible, which further illustrated the effectiveness and universal applicability of the improved algorithm. The reconstruction of knee joint image is shown in Figure 5, and the image processed by SMASH algorithm is obviously clearer.

### 3.2. Analysis of the Results of Different Methods

**Figure 6:**
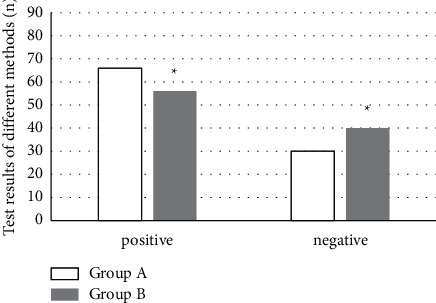
Diagnostic results of different methods for cruciate ligament injury. ^*∗*^Compared with group A, *P* < 0.05.

With arthroscopy as the standard, 66 tests were positive and 30 tests were negative. According to the MRI diagnosis results based on K-space data reconstruction algorithm, 56 tests were positive and 40 tests were negative; the difference between the two groups was statistically significant, *P* < 0.05 (Figure 6).

**Figure 7:**
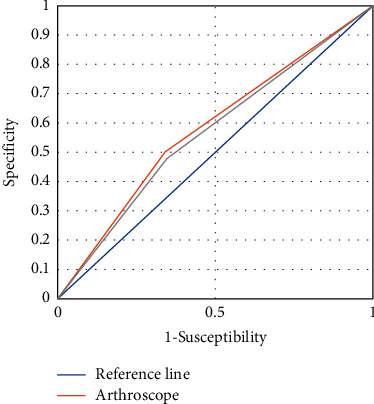
ROC curve.

The two examination methods were plotted as ROC curves, and the area under the ROC curve for arthroscopy was 0.617, standard error 0.7, 95% confidence interval of area (0.766, 0.957) (0.408, 0.716), sensitivity 0.603, and misdiagnosis rate 0.149; the area under ROC curve of MRI detection based on K-space reconstruction algorithm was slightly lower than that of arthroscopy (Figure 7).

### 3.3. Comparison of Anatomic Parameters

**Figure 8:**
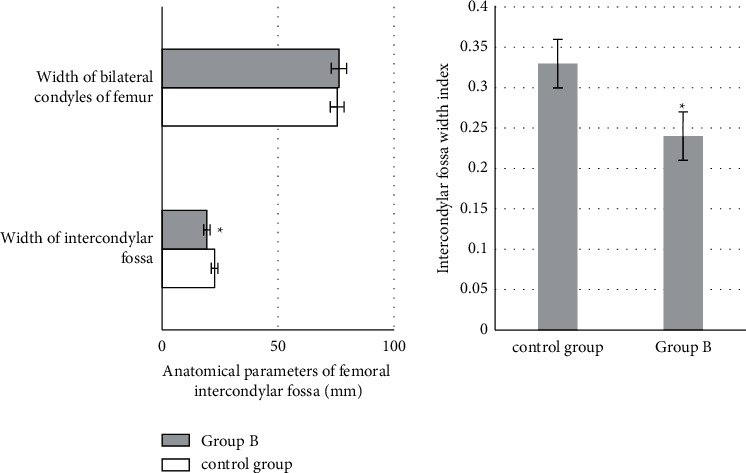
Measurement results of related anatomical parameters of femoral intercondylar fossa. ^*∗*^Compared with the control group, *P* < 0.05.

**Figure 9:**
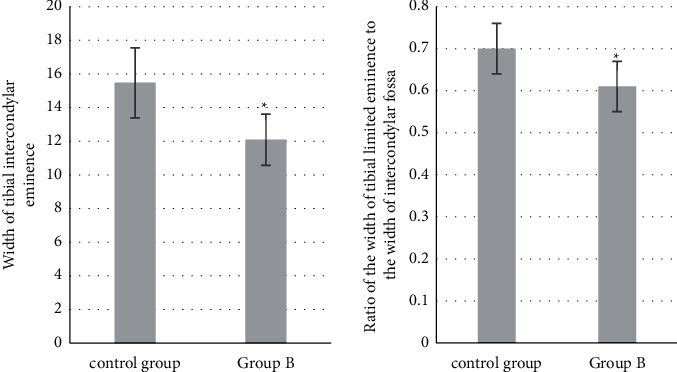
Measurement results of anatomical parameters related to tibial intercondylar eminence. ^*∗*^Compared with the control group, *P* < 0.05.

There was no significant difference in the width of femoral condyle between the two groups (*P* > 0.05). The width of intercondylar fossa, intercondylar fossa width index, and the ratio of tibial intercondylar eminence width to intercondylar fossa width in group B were lower than those in the control group (*P* < 0.05) (Figures 8 and 9).

## 4. Discussion

In the past, the SOS algorithm used to synthesize the reconstructed images of each phased array coil with equal weight greatly affected the final image quality. In addition, it could not well suppress the external noise, and the final image had problems such as signal deviation and reduced SNR [18]. Aiming at remedying its shortcomings, the K-space-based SMASH reconstruction algorithm is applied. First, the reconstructed images of each phased array coil are denoised using a smoothing filter, and then the final images are synthesized by using the coil sensitivity as a weight. The experimental results show that the SNR of the SMASH algorithm is higher than that of the SOS algorithm; there is almost no background noise in the difference image between the synthesized image and the reference image of the SMASH algorithm, and the error image is almost invisible. This shows that the SMASH algorithm can improve the image quality, has superiority, can effectively reduce the impact of corrupted data on the final synthetic image, can effectively eliminate the artifacts in the image, and can improve the image SNR.

The patients were examined by MRI based on SMASH algorithm. The results of arthroscopic examination showed that the number of positives was 66 and the number of negatives was 30. The results of MRI diagnosis based on K-space data reconstruction algorithm showed that the number of positives was 56 and the number of negatives was 40. The difference between the two groups had statistical significance (*P* < 0.05). After the experiment, the reasons were considered and summarized in combination with the relevant literature: the direction during MRI scanning was not parallel to the ACL, and the number of upper layers of section imaging was small; the hematoma of the surrounding tissues of patients in the acute phase would cause artifacts; the volume effect caused artifacts, affecting the judgment [19].

The ACL is an important structure to maintain the stability of the knee joint. After injury, the biomechanical structure of the knee joint is disordered, and the knee joint shows anterior instability, which leads to the injury of intra-articular cartilage, meniscus, and other structures and accelerates the aging and degeneration of the knee joint. Therefore, it is very important to identify the risk factors of ACL injury for its early prevention and treatment. The results showed that there was no significant difference in femoral bicondylar width between the two groups (*P* > 0.05). The intercondylar fossa width, intercondylar fossa width index, ratio of tibial intercondylar eminence width to intercondylar notch width in group B were lower than those in the control group (*P* < 0.05). Some studies have suggested that the intercondylar fossa can be used as a valuable predictor to predict ACL injury, and the intercondylar fossa width index is weakly correlated with ACL injury [20]. This reveals that the poorer the fit between the tibial intercondylar eminence and the femoral intercondylar fossa is, the more likely it is to lead to ACL rupture, but the specific mechanism of effect needs to be clarified by the sample size and biomechanical studies on this aspect.

## 5. Conclusion

MRI based on K-space data reconstruction algorithm was applied to diagnose ACL injury of the knee and assess the risk factors for its occurrence. The results showed that compared with the traditional SOS algorithm, the K-space-based SMASH algorithm could effectively improve the image quality, reduce the impact of corrupted data on the final synthetic image, and improve the image SNR. Compared with the arthroscopy results, the MRI based on the K-space data reconstruction algorithm was found to have certain reference value for ACL injury of the knee, but its accuracy needed to be further improved. The ratio of tibial intercondylar augmentation width to femoral intercondylar notch width could be used to evaluate the matching of tibial intercondylar augmentation and femoral intercondylar notch in clinical practice, so as to evaluate the risk of ACL rupture. However, due to the limitation of conditions, the sample size included in this experiment is small, and the accuracy of some results needs to be further confirmed.

## Data Availability

The data used to support the findings of this study are available from the corresponding author upon request.
